# Unanticipated Difficult Airway During Elective Surgery: A Case Report and Review of Literature

**DOI:** 10.7759/cureus.32996

**Published:** 2022-12-27

**Authors:** Nickhil Rugnath, Lindsay E Rexrode, Lakshmi N Kurnutala

**Affiliations:** 1 Anesthesiology, University of Mississippi Medical Center, Jackson, USA; 2 Anesthesiology and Perioperative Medicine, University of Mississippi Medical Center, Jackson, USA

**Keywords:** difficult airway algorithm, tracheostomy, subglottic stenosis, failed intubation, difficult airway

## Abstract

Difficult airway during anesthesia is responsible for several cases of morbidity and mortality worldwide, especially when it is unanticipated. Patients with either history of or with predictive factors of a difficult airway show better outcomes since all preventative measures will ensure patient safety. Approximately 30% of all deaths attributed to anesthesia are related to unsuccessful intubation. In this article, we discuss a patient who had a tracheostomy following an unanticipated difficult airway with undiagnosed subglottic stenosis and also reviewed the current literature on the difficult airway.

## Introduction

Difficult airway during anesthetic management is one of the significant fears anesthesiologists have when attempting to perform general anesthesia. It can lead to severe implications, including airway trauma, brain hypoxia, and cardiopulmonary arrest, which could result in death if not treated quickly [1]. Early recognition and decisive decision-making are vital to reduce the potential of serious injury. Anesthesiologists often face the dilemma involving the choice of anesthesia to be performed, with each preference having its pros and cons regarding the efficacy and safety of the patient. The primary concern for anesthesiologists is the unpredictable occurrence rate of difficult airway cases, as confounding variables provide a small indication of the potential risk of a difficult airway case occurring [2]. Obesity, for example, markedly increases the probability of airway complications, but they are often unpredictable and require action within minutes [1]. The anesthesiologist may encounter difficulty with anesthesia administration in mask ventilation or placement of supraglottic airway (SGA), SGA ventilation, laryngoscopy, tracheal intubation, or involvement of both ventilation and intubation [3-5]. Airway complications require attentiveness and efficiency on an individual and institutional level. Introducing innovative techniques and failed technique analysis is the key to improving the case outcomes and becoming one step closer to eradicating avoidable causalities involving difficult airway cases [1,6].

## Case presentation

A 63-year-old male (BMI- 33.7 kg/m2) was scheduled for a diagnostic laparoscopy. The patient’s medical history included hypertension, diabetes mellitus, obesity, coronary artery disease, and an s/p pacemaker placement in 2012 for arrhythmia. The patient did not complain of breathing problems at rest or lying down and had no significant past anesthesia history. The patient was seen by an EP (electrophysiology) for pacemaker evaluation, with the recommendation of placing a magnet over the pacemaker when using monopolar surgical cautery. The patient’s fasting blood sugar was 170 mg/dL. The preoperative airway assessment was a Mallampati Class III, thyromental distance (TMD)>3 fingers, a good mouth opening, and normal neck mobility. After standard American Society of Anesthesiologists (ASA) monitors were connected to the patient in the operating room, anesthesia was performed with rapid sequence induction, and intubation was attempted with a Miller #2 blade. The anesthesia provider visualized the glottic opening but could not pass the 8.0 mm endotracheal tube (ETT) beyond the vocal cords. The anesthesia provider then changed to a 7.0 mm ET tube but was still unsuccessful with the intubation passing below the vocal cords. We placed the ET tube above the vocal cords with minimal cuff inflation to continue ventilation and maintain oxygenation. The glidescope visualized the upper glottis and vocal cords but was still unsuccessful in passing the boogie through the 7.0 mm ETT. Next, the fibreoptic bronchoscope (FOB) was introduced into the ET tube but was still unsuccessful in passing beyond the tip of the ETT. The ear, nose, and throat (ENT) surgeon was contacted for airway evaluation while the patient was still ventilated with a 7.0 mm ETT at the vocal cord level.

**Figure 1:**
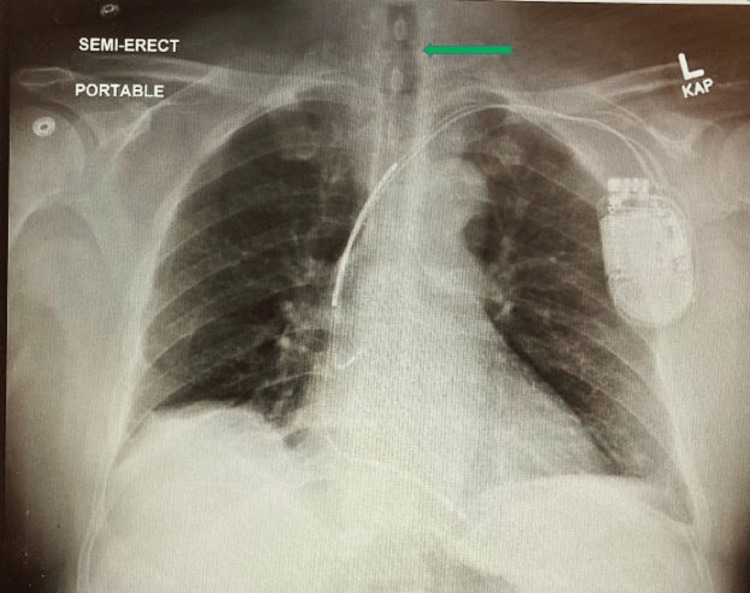
A chest X-ray done in the PACU shows subglottic stenosis with a decreased and irregular air column shown by a green arrow. PACU: Post-anesthesia care unit

ENT surgeon evaluated the airway with a pediatric FOB bronchoscope with an eyepiece, discovering severe subglottic stricture with stenosis. After a discussion with general surgery and an ENT surgeon, the patient was treated with an intraoperative steroid (Dexamethasone 8mg), extubated after meeting extubation criteria, and transported to a post-anesthesia recovery room (PACU). ENT planned to perform a direct laryngoscopy with possible dilation of the stenotic area as an elective procedure later. In PACU, the patient became dyspneic with minimal air entry into the lungs on auscultation, with an SpO2 of 88%-90%. He was treated with racemic epinephrine with a face mask, and a chest X-ray in the PACU showed subglottic stenosis with decreased air column (Figure 1).

Arterial blood gas analysis (ABG) showed PCO2 was 91 mm Hg, and the patient was still tired. The surgery team and ENT were informed and decided to perform an emergency tracheostomy to secure the patent airway. The patient was immediately transferred to the operating room (OR) with Ambu with mask ventilation. In the OR, the patient was ventilated with a face mask (oxygen, air mixture with an inhalation agent), breathing spontaneously. The ENT surgeon performed the tracheostomy and placed an 8.0mm cuffed tracheostomy tube with confirmation of position and bilateral air entry. During the surgical procedure, the patient’s vital signs were stable and met adequate anesthesia goals. The patient was transferred to the medical intensive care unit (MICU) for observation.

## Discussion

**Figure 2:**
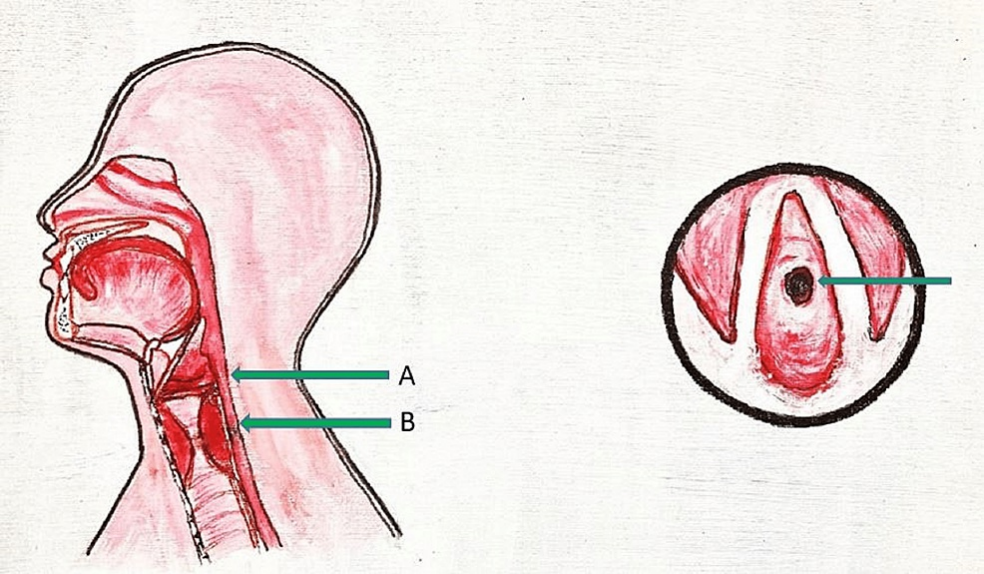
Diagrammatic representation of a sagittal section of subglottic stenosis on the left side (A: Glottis, B: Subglottic stenosis) with a transverse section on the right side with a view from above showing the narrowing of the Subglottis (Green arrow). Painting credit Lakshmi N Kurnutala M.D.

Subglottic stenosis is a constriction below the vocal cords and above the trachea, known as the subglottic region [7] (Figure 2).

**Figure 3:**
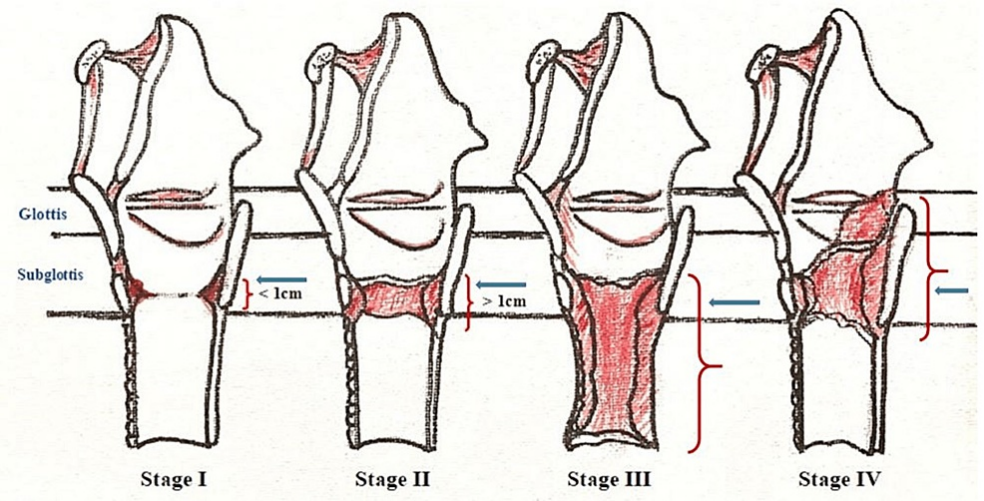
McCaffrey classification of development of subglottic stenosis. Green arrows in each stage show involvement of subglottis. Painting credit Lakshmi N Kurnutala M.D.

It involves cricoid cartilage narrowing, the only complete ring in the airway of humans. Subglottic stenosis typically ensues after incidental larynx scarring, but research has also concluded that other factors could play an additional role [8]. Subglottic stenosis comes in two forms: acquired subglottic and congenital subglottic stenosis [9]. Acquired subglottic stenosis develops after birth. The most common causes of acquired subglottic stenosis are infection, trauma, and long periods of intubation and ventilation (typical of respiratory problems). Congenital subglottic stenosis is present at birth, in contrast to acquired subglottic stenosis. Congenital subglottic stenosis stems from the inability of normal airway cartilage formation before birth, usually associated with certain genetic conditions and syndromes [7-10]. Based on the diameter of subglottis in cross-section, Mayer-cotton classified subglottic stenosis into grade I (no obstruction - 50% obstruction), grade II (51%-70%), grade III (71%-99%) and grade IV (no detectable lumen [9]. McCaffrey classified the development of subglottic stenosis into four stages, with an increase in subglottic length of involvement [9] (Figure 3).

Treatment for subglottic stenosis is divided into nonsurgical management like Heliox therapy, dilation, and stenting, interventional bronchoscopy with laser therapy; surgical management includes an open surgery with laryngotracheal reconstruction surgery (LTR) or partial cricotracheal resection (CTR). LTR and CTR correct the stenosis by achieving decannulation, which is tracheostomy tube removal [5]. Studies have shown that 96% of children with subglottic stenosis achieve decannulation when undergoing a posterior graft LTR, which proves to be a very successful treatment and management of subglottic stenosis [9].

**Figure 4:**
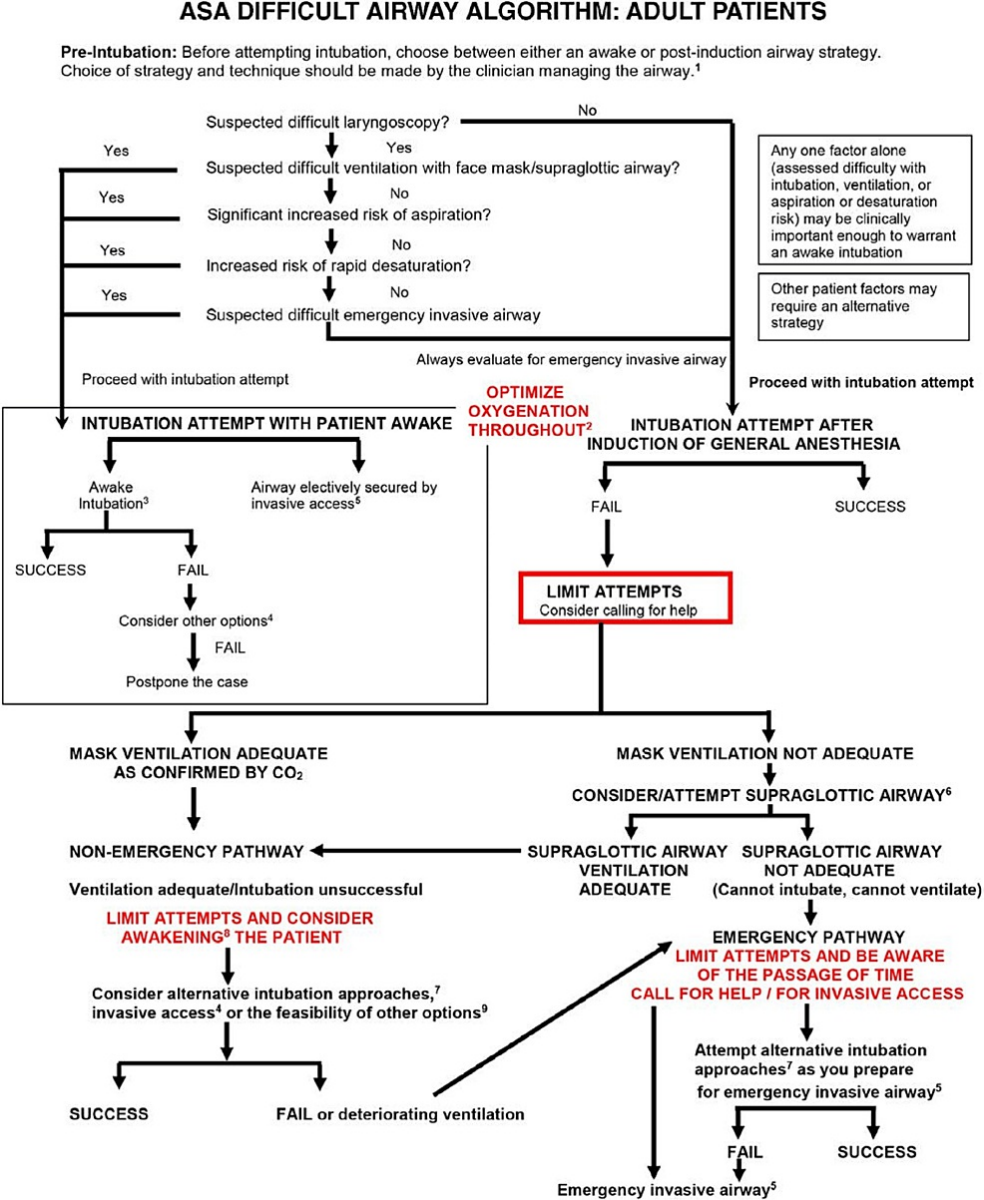
ASA Difficult airway algorithm: Adult patient. Adopted from 2022 American Society of Anesthesiologists Practice Guidelines for Management of the Difficult Airway. ^1^The airway manager’s choice of strategy and techniques based on their previous experience; available resources ^2^Low- or high-flow nasal cannula, head elevated position throughout the procedure. Noninvasive ventilation during preoxygenation. ^3^Awake intubation techniques include flexible bronchoscopes, videolaryngoscopy, direct laryngoscopy, combined techniques, and retrograde wire-aided intubation. ^4^Other options, but are not limited to, alternative awake technique, awake elective invasive airway, alternative anesthetic techniques, induction of anesthesia (if unstable or cannot be postponed) with preparations for emergency invasive airway, and postponing the case without attempting the above options. ^5^Invasive airway techniques - surgical cricothyrotomy, needle cricothyrotomy with a pressure-regulated device, large-bore cannula cricothyrotomy, or surgical tracheostomy. Elective invasive airway techniques include the above and retrograde wire–guided intubation and percutaneous tracheostomy. Also consider rigid bronchoscopy and ECMO. ^6^Consideration of size, design, positioning, and first- versus second-generation supraglottic airways may improve the ability to ventilate. ^7^Alternative difficult intubation approaches include but are not limited to video-assisted laryngoscopy, alternative laryngoscope blades, combined techniques, intubating supraglottic airway (with or without flexible bronchoscopic guidance), flexible bronchoscopy, introducer, and lighted stylet or light wand. Adjuncts that may be employed during intubation attempts include tracheal tube introducers, rigid stylets, intubating stylets, tube changers, and external laryngeal manipulation. ^8^Includes postponing the case or postponing the intubation and returning with appropriate resources. ^9^Other options include, but are not limited to, proceeding with the procedure utilizing a face mask or supraglottic airway ventilation. Pursuit of these options usually implies that ventilation will not be problematic.

Of all subglottic stenosis cases, 90% of subglottic stenosis cases result from endotracheal intubation, with 0.9%-8.3% of subglottic stenosis cases arising following difficult intubation [10]. In rare cases like this one, the cause of the subglottic stenosis is unknown, termed idiopathic subglottic stenosis. The unpredictability of idiopathic subglottic stenosis introduces the question, “How do we become effectively prepared for situations like this?” [10,11]. To effectively manage unpredictable cases and minimize potential implications, every aspect of the medical team must be integrative and maintain open communication to ensure productive analysis in a multifaceted approach [3]. The American Society of Anesthesiologists (ASA) difficult airway guidelines 2022 provide anesthesiologists guidance for efficient preparation and management of patients with difficult airways [1] (Figure 4).

These are readily available preparatory interventions to assist in a difficult airway management case should it arise [11,12]. These interventions are: (a) the availability of equipment for airway management; (b) informing the patient of a known/suspected airway difficulty; (c) pre-oxygenation; (d) patient positioning; (e) sedative administration; (f) local anesthesia; (g) supplemental oxygen during difficult airway management; (h) patient monitoring; and finally (i) human factors [1,6]. These guidelines put anesthesiologists in the best position possible to handle difficult airway patients, preventing the likelihood of any severe implications.

Although these guidelines are in place, more must be done to efficiently and effectively ensure the proper management of difficult airway patients. One example is the level of communication among the physicians described in this case. Effective communication is a vital component of successfully addressing this difficult airway patient. For example, the collaboration between the anesthesiologists, anesthesia providers (CRNAs, residents), and the general surgery team with the ENT surgeon allowed for effective treatment and management of this case, ensuring the optimal outcome [13,14]. Collaboration like this should be implemented more often in medical practice today, ensuring that the practice of medicine is consistently performed with a “team” approach. Without this approach, the possibilities of implications are increased, not only putting the patient at risk but also increasing the chances of mismanagement of the difficult airway, which could be potentially fatal [5].

## Conclusions

The unanticipated difficult airway is a nightmare scenario for anesthesiologists; it relates to patient factors, associated clinical conditions, anesthesiologist skills, and preparation for airway management. In situations of a difficult airway with unknown clinical conditions like subglottic stenosis, the anesthesiologist may need to act as a team leader and coordinator of airway care. Management of difficult airways should always require a “team” approach, ensuring that the diagnosis is confirmed from multiple sources and collaborations involving every clinical personnel to guarantee the best possible outcome for the patient at all times.
